# Precision dosing of amikacin in term neonates using pharmacometric approach

**DOI:** 10.1038/s41390-025-04044-7

**Published:** 2025-04-10

**Authors:** Saikumar Matcha, Jaya Shree Dilli Batcha, Arun Prasath Raju, Bhim Bahadur Chaudhari, Sudheer Moorkoth, Surulivelrajan Mallayasamy, Leslie E. Lewis

**Affiliations:** 1 Research Scholar, Department of Pharmacy Practice, Manipal College of Pharmaceutical Sciences, Manipal Academy of Higher Education, Manipal, India; 2 Titus Family Department of Clinical Pharmacy, Alfred E. Mann School of Pharmacy and Pharmaceutical Sciences, Los Angeles, CA USA; 3 Center for Pharmacometrics, Manipal Academy of Higher Education, Manipal, India; 4 Research Scholar, Department of Pharmaceutical Quality Assurance, Manipal College of Pharmaceutical Sciences, Manipal Academy of Higher Education, Manipal, India; 5 Professor & Head, Department of Pharmaceutical Quality Assurance, Manipal College of Pharmaceutical Sciences, Manipal Academy of Higher Education, Manipal, India; 6 Professor & Head, Department of Pharmacy Practice, Manipal College of Pharmaceutical Sciences, Manipal Academy of Higher Education, Manipal, India; 7 Professor & Head, Department of Pediatrics, Kasturba Medical College, Manipal Academy of Higher Education, Manipal, India

## Abstract

**Background:**

Maintaining amikacin concentrations within a specific therapeutic window is crucial to avoid sub-therapeutic or toxic levels. This study aimed to design a dosing nomogram for amikacin in neonates using a Population Pharmacokinetic (PopPK) modeling approach.

**Methods:**

PopPK model was developed using 101 amikacin concentrations from 80 neonates and validated using model diagnostics, and empirical Bayesian forecasting was performed. Pharmacokinetic profiles were simulated for virtual subjects with a range of covariates to identify suitable dosage regimens. Dosage regimens with the highest probability for the target group were selected to design the dosing nomogram.

**Results:**

A two-compartment PK model best described the study data. Body weight (WT), serum creatinine (SCR), and post-natal age (PNA) affected the clearance of amikacin. The model predictions are with less than 15% absolute prediction error. WT and SCR were divided into five groups each, with each group repeated for every week of PNA for four weeks for dosing nomogram development.

**Conclusion:**

A PopPK model was developed and successfully-predicted concentrations in the study population. This model was used to develop a nomogram considering significant covariates like WT, SCR, and PNA. The proposed dosing nomogram can assist clinicians in developing individualized dosage regimens.

**Impact:**

- Population pharmacokinetic (PopPK) models for amikacin in term neonates were developed using clinical data from an Indian clinical setting and successfully-predicted the amikacin concentrations for the study population.
- Pharmacokinetic simulations with virtual subjects were used to calculate the probability of target attainment for different dosing regimens.
- The proposed dosing nomogram can potentially assist clinicians in designing optimal amikacin dosage regimens for neonates.

## Introduction

Neonates undergo the most intense physiological changes compared to other life stages.^1^ Their immune systems are not fully mature, making them vulnerable to infections.^2^ Infections are associated with 36% of the approximately 4 million neonatal deaths per year,^3^ with the most of these infections ( > 90%) being bacterial, followed by fungal infections (4%).^4^

In the Neonatal Intensive Care Unit (NICU), beta-lactam antibiotics, aminoglycosides, and vancomycin are the most prescribed antibiotics.^5^ Gentamicin, an aminoglycoside, is the first choice for infections caused by Gram-negative bacterial strains (GNBS). Combination therapy with gentamicin and ampicillin covers most infections caused by GNBS and Gram-positive bacterial strains (GPBS).^5,6^ However, the World Health Organization (WHO) has raised concerns about antibiotic resistance to ampicillin and gentamicin.^7^ Consequently, ampicillin has been largely replaced by piperacillin-tazobactam, and amikacin has replaced gentamicin as the first-line antibiotic in the South-East Asia region and African regions.^8^ The efficient bactericidal activity, cost-effectiveness, and broad-spectrum coverage of amikacin make it the first-line agent for managing neonatal sepsis.^9,10^

Amikacin, a semisynthetic analog of kanamycin, was developed to overcome aminoglycoside resistance caused by aminoglycoside-modifying enzymes.^11^ It is administered as an intravenous (IV) infusion, with dosing in neonates starting at 10 mg/kg per day and potentially increasing to 20 mg/kg per day under certain conditions.^12^ Amikacin exhibits a concentration-dependent killing mechanism, requiring peak concentrations to be 8 times higher than the minimum inhibitory concentration (MIC) of the targeted bacteria.^13^ Dosage regimens aim to achieve peak and trough concentrations within the ranges of 24–35 mg/L and 2–5 mg/L, respectively.^14^ As a renally eliminated drug, amikacin can cause nephrotoxicity and ototoxicity at higher concentrations.^13^ Therefore, a thorough understanding of the clinical pharmacokinetics (PK) of amikacin is essential for designing optimal dosage regimens.

Traditional PK studies are challenging to conduct in special populations like neonates. Population pharmacokinetics (PopPK) is an effective approach to understanding the PK behavior of drugs in these populations.^15^ Unlike traditional PK studies, PopPK studies can describe PK characteristics with minimal sample collection at various times.^16^ PopPK model simulations help to explore various dosage regimen scenarios. Concentration data from therapeutic drug monitoring (TDM) can be used to estimate individual PK parameters,^17^ which assists clinicians in designing model-informed precise dosage regimens.^18^ This study aimed to assess the empirical Bayesian performance of a PopPK model to assist TDM in clinical settings. Additionally, the study aimed to develop a PopPK model and propose a dosing nomogram for the initial dose of amikacin in neonates.

## Methodology

### Patient recruitment and data collection

This prospective observational longitudinal study was approved by the institutional ethics committee (ECR/146/Inst/KA/2013/RR-19; IEC:558/2019). Demographic and clinical data were collected from medical records using a predesigned data collection form. Term neonates prescribed with amikacin were included, while clinically unstable neonates were excluded. Informed consent was obtained from a parent or guardian for all participants.

### Collection of blood samples and quantification of drug levels

Blood samples of 0.3 ml were collected at convenient time points, during regular laboratory investigations. Amikacin concentrations were quantified using an in-house developed liquid chromatography with tandem mass spectrometry (LC-MS/MS) bioanalytical method.^19^ This method was linear over the range of 0.5–100 mg/L for amikacin concentrations, with an R² value of 0.99, precision around 6%, and accuracy between 93% and 98%.

### PopPK model building

#### Base model

One- and two-compartment models were tested to select the base model. Various error model structures, including additive, proportional, and combined models, were evaluated. Between-subject variability (BSV) was tested on all PK parameters, and significant ones were included. Parameters were assumed to have a log-normal distribution, so BSV was modeled in exponential terms. Subject fits, goodness-of-fit (GOF) plots, and statistical measures including the -2 log-likelihood value (-2LL)/Objective function value (OFV), Akaike information criterion (AIC), Bayesian information criterion (BIC), and chi-square test were used to select the better model. All analyses were performed using the Pumas package within the Julia computing environment (version 1.6).^20^

#### Covariate selection

Covariates with meaningful pharmacological and physiological relations to the parameters were listed and tested. Power and exponential functions were used to describe the covariate effects for continuous covariates, while a proportional shift model was used for categorical covariates. A forward addition and backward deletion approach identified significant covariates. decrease in OFV by 3.84 points were considered as significant for forward addition and 6.63 for backward deletion. For chi square test, *p* < 0.05 was considered for forward addition and *p* < 0.01 was considered for backward deletion. Individual fits and GOF plots were inspected at each step.

#### Model evaluation

The final model was evaluated using Subject fits, GOF, OFV, -2LL value, chi-square test, visual predictive check (VPC, *n* = 500), and bootstrapping procedure (*n* = 1000).

#### Dosing nomogram

Using the final model, virtual subjects were developed for the range of covariates. One hundred virtual subjects were created in each group. PK profiles were generated 100 times for each subject using BSV. Target attainment was tested for each dosage regimen using different combinations from the covariate value range. Target attainment window for amikacin was determined based on a review of the literature. Peak concentrations of 24–35 mg/L and trough concentrations of 2–5 mg/L were considered optimal. Dosage regimens with the highest probability of attaining the target were selected to design the dosing nomogram.

#### Empirical Bayesian prediction

The final model was evaluated for its suitability using empirical Bayesian prediction for implementing TDM. Observed concentrations were used to estimate individual parameters, which were then used to simulate further concentrations. Observed concentrations were compared against individual predicted concentrations to assess model performance. Relative mean individual prediction error percentage (rMIPE%) and relative median absolute individual prediction error percentage (rMAIPE%) were calculated. Predictions falling within 20% and 30% were considered satisfactory for rMIPE% and rMAIPE%, respectively.

1 $${{\rm{rIPE}}} \% =\left(\frac{{Cipred}-{Cobs}}{{Cobs}}\right)* \,100$$

2 $${{\rm{rMIPE}}} \% =\frac{1}{n}{\sum }_{i=1}^{n}\left(\frac{{Cipred}-{Cobs}}{{Cobs}}\right)* \,100$$

3 $${{\rm{rMAIPE}}} \% ={{\rm{median}}}\; {{\rm{of}}}\; |\left(\frac{{Cipred}-{Cobs}}{{Cobs}}\right)* \, 100|$$

In addition, observed concentrations were overlapped on the predicted PK profiles of individual subjects to visually check the predictive performance of the final model.

## Results

**Fig. 1:**
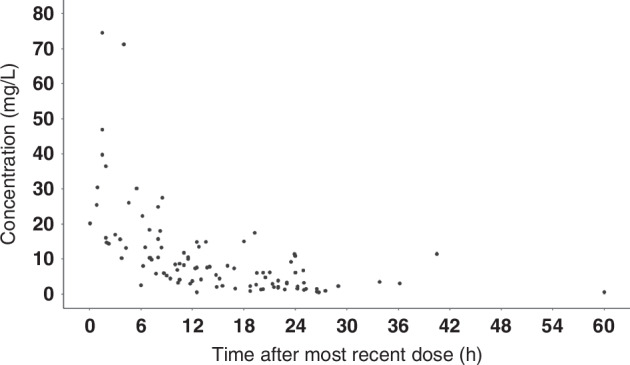
Time after most recent amikacin dose vs concentration plot. Amikacin concentration vs. time after most recent dose plot.

A total of 101 amikacin concentrations were collected from 80 subjects. Patient characteristics and laboratory investigations, such as weight (WT), height (HT), and serum creatinine (SCr), were filled with the most recent available information for each subject if missing at the time of blood sampling. Unusually high or low concentrations were considered outliers and removed from the dataset. The issues faced during data cleaning are detailed in supplementary table 1. Ultimately, 100 concentrations from 78 subjects were used for model building. The median post-natal age (PNA) at the initiation of treatment and at the time of sample collection was 2 and 5 days, respectively. Creatinine clearance (CrCl) was calculated using the Schwartz formula with a constant of *k* = 0.45.^21^ Demographic details of the study population are given in Table 1. Blood samples were collected at random time points, covering most intervals between 0.5 and 24 h after the most recent amikacin dose. The dispersion of sample collection times after the most recent amikacin dose is shown in Fig. 1.

**Table 1:** Demographic details of study population considered for model building.

Characteristic	Median (Range)
PNA at start of amikacin (Days)	1.70 (0.04–21.29)
PNA at sample collection (Days)	5.02 (0.62–27.32)
GA (weeks)	39 (37–42)
PMA at start of amikacin (weeks)	39.20 (37.11–42.22)
BWT (Kg)	2.95 (1.75–4.50)
CWT at start of amikacin (Kg)	2.85 (1.74–4.84)
HT (cm)	48 (40–58)
Dose (mg)	40 (25–110)
Dose (mg/kg)	12.52 (8.23–51.89)
SCr (mg/dL)	0.55 (0.16–1.49)
CrCl (ml/min)	40.98 (12.68–145.0)
No. of observations	100
No. of Subjects	78

*PNA*: Post Natal Age, *GA* Gestational Age, *PMA* Post Menstrual Age, *BWT* Birth Weight, *CWT* Current Body Weight, *HT* Height, *SCr* Serum Creatinine, *CrCl* Creatinine Clearance.

A two-compartment structural PK model best explained the study data. BSV on clearance (CL) and the volume of central compartment (VC) were statistically significant. A proportional residual error model was chosen to explain residual unexplained variability. GOF plots of base model is shown in supplementary Fig. 1. All clinically relevant covariates tested on each parameter are shown in supplementary table 2. A total of 40 models were tested to identify statistically significant covariates. PNA, WT, and SCr were identified as statistically significant covariates on CL during the forward addition step. Although covariance between CL and VC was statistically significant, parameter estimates were not clinically meaningful and hence not considered. Two steps of backward deletion confirmed WT, SCr, and PNA as significant covariates in the final model. The OFV of the base model was 422.24. Incorporating WT as a covariate on CL reduced the OFV to 416.41 and decreased the BSV on CL from 39.2% to 37.8%. Adding SCr as a covariate on CL further reduced the BSV on CL to 33.6% and lowered the OFV to 380.64. The BSV on VC was 41.1% in the final model, compared to 48.3% in the base model. The structure of the final model is given below:

4 $${{{\rm{CL}}}}_{{{\rm{i}}}}=0.064* {{Exp}}^{{WT}* 0.308}* {\left(\frac{{SCrmean}}{{SCr}}\right)}^{0.397}* {{EXP}}^{{eta}{{\rm{\_}}}{cl}}$$

5 $${{{\rm{VC}}}}_{{{\rm{i}}}}=1.281* {{EXP}}^{{eta}\_{vc}}$$

6 $${{{\rm{VP}}}}_{{{\rm{i}}}}=0.618$$

7 $${{{\rm{Q}}}}_{{{\rm{i}}}}=0.055$$

Where i represents individual estimate whereas, CL: Clearance; WT: Weight; SCr: Serum Creatinine; VC: Volume of Central Compartment; VP: Volume of Peripheral Compartment; Scrmean; Q: Intercompartmental Clearance; SCrmean: average SCr level of population at that age; BSV on CL and VC was estimated as 33.6% and 41.1% respectively. Scrmean was calculated using the below formula.^22^

8 $${{\rm{SCrmean}}}=-0.02324-0.14545\times \log ({{\rm{PNA}}})+0.26964\times {{{\rm{PNA}}}}^{0.5}$$

**Fig. 2:**
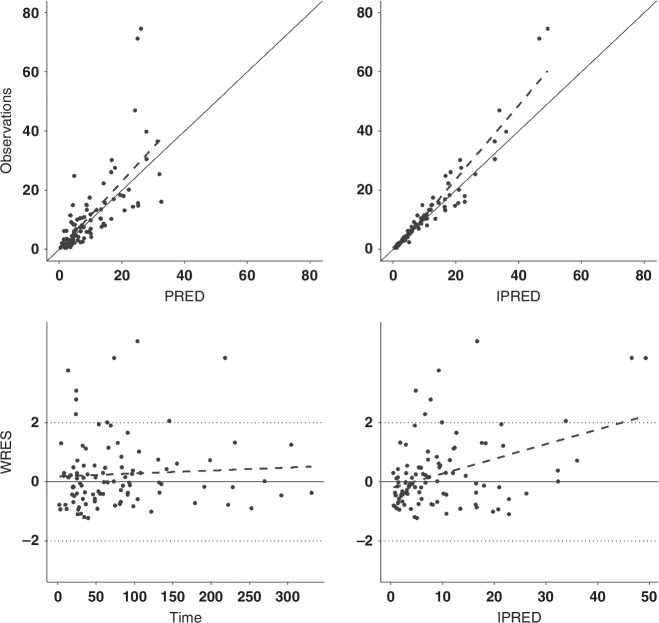
Goodness of fit plots of final model. Solid Line represents the identity line and dashed line illustrates the trend.

**Fig. 3:**
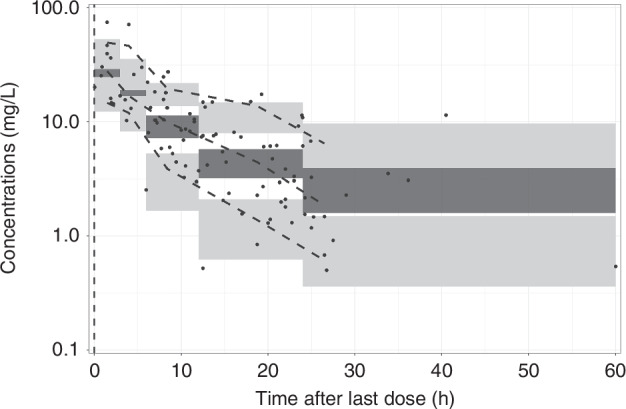
Visual Predictive check of final model. Black dot—Observations balck dashed lines—80% interval and median of the observations. Dark gray shaded area—95% CI of the median prediction, light gray shaded area = 95% CI of the 10th and 90th prediction interval.

Scatter plots were inspected to check the GOF to the data, as shown in Fig. 2. Bootstrap runs were 100% successful, with results shown in Table 2, VPCis shown in Fig. 3.

**Table 2:** Parameter and bootstrap estimates of the final model.

Parameters	Typical estimates (SE) [% shrinkage]	Median (95% CI) for 1000 bootstrap replicates
TVCL (L/h)	0.064 (0.022)	0.064 (0.033–0.157)
TVVC (L)	1.281 (0.287)	1.279 (0.371–1.840)
TVQ (L/h)	0.055 (0.025)	0.055 (0.019–0.375)
TVVP (L)	0.618 (0.325)	0.615 (0.248–1.357)
BSV_cl (%CV)	33.6 [19.7]	32.5 (15.2–46.3)
BSV_vc (%CV)	41.1 [35.2]	40.8 (8.1–65.1)
Proportional residual error (%CV)	30.9 [31.0]	31.1 (13.2–45.6)
Wt_cl	0.308 (0.116)	0.301 (0.07–0.514)
SCr_cl	0.397 (0.109)	0.385 (0.241–0.593)

*SE* Standard Error, *CI* Confidence Interval, *TVCL* Typical Value of Clearance, *TVVC* Typical Value of Volume of Central compartment, *TVQ* Typical Value of Intercompartmental clearance, *TVVP* Typical Value of Volume of Peripheral compartment, *BSV_cl* Between Subject Variability on Clearance, *BSV_vc* Between Subject Variability on Volume of Central compartment, *WT_cl* Magnitude of Weight on Clearance, *SCr_cl* Magnitude of Serum Creatinine on Clearance.

Virtual subjects were developed using combinations of covariates (WT & SCr). The WT and SCr ranges considered were 2–4.5 kg and 0.15–1.50 mg/dL, respectively. WT and SCr were divided into five subgroups each, forming 25 subgroups. Each subgroup was repeated for every week of the neonatal period. Dosage regimens from 7.5–20 mg/kg at 6–48 h intervals were tested on each subgroup. Dosage regimens with the highest probability of target attainment were chosen to develop a dosing nomogram, as shown in Table 3. Across all dosage regimens, approximately 50% of peak concentrations fell within the target window. Around 30% of the peaks exceeded the target window, while approximately 20% were below it. For trough concentrations, approximately 50% were within the target window, 20% were above it, and 30% were below target range.

**Table 3:** Dosing nomogram for amikacin in term neonates.

WT	2.00–2.49	2.50–2.99	3.00–3.49	3.50–3.99	4.00–4.50
SCR					
PNA: 1–7 days					
0.15–0.29	17 q 24H	15 q 21H	12 q 18H	11 q 15H	9 q 12H
0.30–0.49	17 q 30H	13 q 24H	12 q 24H	10 q 18H	10 q 18H
0.50–0.74	16 q 36H	13 q 30H	12 q 30H	10 q 24H	9 q 21H
0.75–0.99	16 q 42H	13 q 36H	12 q 30H	10 q 24H	9 q 24H
1.00–1.50	16 q 48H	13 q 42 H	12 q 36H	10 q 30H	9 q 24H
PNA: 8–14 days					
0.15–0.29	18 q 21H	15 q 21H	13 q 15H	11 q 12H	11 q 12H
0.30–0.49	17 q 24H	13 q 18H	12 q 18H	11 q 15H	11 q 15H
0.50–0.74	16 q 30H	13 q 24H	12 q 24H	10 q 18H	9 q 15H
0.75–0.99	17 q 36H	14 q 30H	12 q 24H	10 q 21H	9 q 18H
1.00–1.50	17 q 42H	14 q 36H	12 q 30H	10 q 24H	9 q 21H
PNA: 15–21 days					
0.15–0.29	18 q 18H	15 q 15H	13 q 12H	13 q 12H	11 q 9H
0.30–0.49	18 q 18H	14 q 18H	13 q 18H	12 q 15H	10 q 12H
0.50–0.74	17 q 30H	14 q 24H	12 q 21H	11 q 18H	10 q 15H
0.75–0.99	16 q 30H	13 q 24H	12 q 21H	10 q 18H	9 q 15H
1.00–1.50	16 q 36H	13 q 30H	12 q 24H	10 q 21H	9 q 18H
PNA: 22–28 days					
0.15–0.29	17 q 15H	16 q 15H	14 q 12H	14 q 12H	11 q 9H
0.30–0.49	19 q 18H	15 q 18H	13 q 15H	11 q 12H	11 q 12H
0.50–0.74	17 q 24H	14 q 21H	12 q 18H	11 q 15H	10 q 12H
0.75–0.99	16 q 24H	14 q 24H	13 q 21H	11 q 18H	10 q 15H
1.00–1.50	16 q 30H	14 q 30H	12 q 24H	11 q 21H	10 q 18H

Units: WT—kg, SCR—mg/dL, dose—mg/kg, dosing interval—hours.

A total of 17 subjects with more than one concentration were considered for empirical Bayesian prediction. The model predicted the second concentrations with 2.88% and 15.23% of rMIPE and rMAIPE%, respectively.

## Discussion

Understanding the PK of drugs in neonates is challenging due to the difficulty of conducting clinical trials in this population.^23^ PopPK modeling is an effective approach to study the PK behavior of drugs with sparse samples in vulnerable populations.^16^ Ten PopPK studies on amikacin in neonates have been published,^14,24–32^ based on clinical data, these studies recommended dosage regimens for their respective populations. However, most of the developed models were not validated using external datasets, highlighting the need for assessment in different clinical settings.

Our study identified a two-compartment structural model for amikacin PK, consistent with recently published literature. Reported CL values in neonates range from 0.031 to 0.23 L/h, with studies using two-compartment models reporting CL between 0.0493 and 0.093 L/h. The CL estimated in our study, 0.064 L/h, falls within this range. Similarly, the reported VC for neonates ranges from 0.316 to 2.94 L, with studies using two-compartment models reporting VC values between 0.637 and 1.03 L. Our study estimated a slightly higher Vc of 1.281 L, which, although outside the two-compartment range, remains within the overall reported range. The reported VP ranges from 0.478 to 1.03 L, with our estimate of 0.618 L falling within this range. Lastly, Q values in the literature range from 0.02 to 0.415 L/h, and our estimate of 0.055 L/h is consistent with this range.^14,24–32^ Most studies identify WT, SCr, PNA as covariates influencing CL, with WT also affecting VC. In our study, we successfully estimated the covariate effects on CL but could not identify significant covariate effects on VC. This limitation may be attributed to the small number of samples per patient and the lack of sufficient samples in the 0–2 h post-dose window, which likely impacted covariate identification for VC.^13,14,25,27–30^

In our previous study, we evaluated the predictive performance of all published models using data from our neonatal setting.^33^ The two-compartment model developed by Illamola et al. ^30^ demonstrated better predictive performance compared to other models. Illamola et al. identified WT and CrCl as covariates for CL and WT for the VC. Our model identified WT, SCr, and SCrmean derived from PNA as covariates for CL. Including PNA allows the incorporation of renal maturation (RM), which is directly proportional to PNA and gestational age (GA).^34^ Adding GA or post-menstrual age (PMA) as covariates did not improve model performance, likely because our study population consisted of term neonates, reducing the impact of GA/PMA on PK parameters. The distribution of PNA from 1 to 28 days allowed us to estimate the effect of PNA on CL.

Guido et al. ^29^ also included SCr as a covariate, but their model predictions were not precise in our previous study.^33^ The inclusion of SCr on the elimination rate constant (Ke) and the lack of other relevant covariates like PNA and WT might have contributed to their model’s poor performance. Most other studies included PMA or PNA as a covariate for CL along with CWT or BWT but did not incorporate SCr or CrCl, potentially leading to poor model performance. The US FDA recommends monitoring SCr levels and considering renal function (RF) when designing dosage regimens.^12^ Both our study and Illamola et al.‘s model included RF as a covariate for CL. Our model also included PNA to account for RM.

Illamola et al., De Cock et al., and Smits et al. proposed dosing regimens based on their findings. However, De Cock et al. and Smits et al. did not consider RF in their nomograms. Illamola et al. suggested two dosage regimens based on WT–PNA and WT–CrCl. Our study considered WT, SCr, and PNA to develop a dosing nomogram. We formed 100 subgroups based on covariate combinations, using narrow intervals for covariates to help clinicians develop individualized therapy. We conducted PK-PD simulations with target peak and trough ranges of 24–35 mg/L and 2–5 mg/L, respectively. The potential MIC required for amikacin’s antibacterial activity is 2–4 mg/L. Our simulations targeted a peak concentration of ~30 mg/L and a trough concentration of ~3 mg/L.

Approximately 50% of simulated peak and trough concentrations were within the targeted therapeutic range, with the remainder distributed equally above and below the therapeutic range. We recommend this dosing nomogram for initial dose selection, with trough level estimations after the first dose to estimate individual CL and PK parameters, aiding in developing subsequent doses. Empirical Bayesian predictions for 17 subjects with more than one concentration showed satisfactory performance, predicting second and third concentrations. The developed model can be utilized to create individualized dosage regimens.

There are some limitations in this study, including missing SCr and WT values filled from the most recent values for the same patient. SCr values of neonates in the first two days of life are influenced by maternal creatinine levels. Therefore, the utility of this dosing nomogram in the first two days of life needs further validation. A prospective evaluation of the proposed dosing nomogram is planned for future studies, and testing of dosing nomogram in different settings and external evaluation of PoppK model is highly encouraged.

## Conclusion

A two-compartment PK model explained the amikacin profile better than other models. WT, SCr, and SCrmean derived from PNA significantly affected amikacin CL. The final model was used to simulate concentrations based on different dosage regimens. The dosing nomogram, chosen based on simulation profiles with the highest probability of attaining the target range, was designed to achieve peak and trough concentrations of 24–35 mg/L and 2–5 mg/L, respectively. The proposed dosing nomogram can be used to determine the initial dose of amikacin for neonates. TDM is recommended after the first dose, and the developed PopPK model can be used to design individualized therapy.

## Supplementary information

Supplementary Material

## Data Availability

Data is available from the corresponding author on the reasonable request.

## Code availability

This work has used the PUMAS-AI software, code is available from corresponding author on request.
