# Supplementary material for: Precision dosing of amikacin in term neonates using pharmacometric approach
Source: Pediatr Res. 2025 Apr 10;98(3):936–41. doi: 10.1038/s41390-025-04044-7 (PMC12507653; doi:10.1038/s41390-025-04044-7)
Supplement: Supplementary file 1 — Supplementary Material [file 41390_2025_4044_MOESM1_ESM.pdf]

Supplementary Table S1: Issues addressed while data cleaning.

Issue	No. of subjects	Adjustment done
Time of birth missing	3	Considered as 12:00 pm
GA missing	11	Filled with 39 weeks
SCr missing	195 rows	Filled with the recent value from same subject
SCr completely missing	12	Filled with SCr predictions (Wang et al., 2019)<br>$-0.02324 - (0.14545 * \log(PNA)) + 0.26964 * (PNA^{0.5})$
CWT missing	163 rows	Filled with the recent value from same subject
CWT completely missing	16 subjects	Filled with BWT
HT missing	1	Filled with median (48 cm)
Unusual concentrations	1 observation	Removed (DV = 154 mg/L)
All concentrations were missing in ID	1	Removed subject

GA: Gestational Age; SCr: Serum Creatinine; CWT: Current Body Weight; BWT: Birth Weight; HT: Height;

Supplementary Table S2: Clinically relevant covariates tested on each parameter

Parameter	Covariates
**CL**	BWT, PNA, GA, PMA, WT, HT,SCr, CrCl
**VC**	BWT,PNA, GA, PMA,WT, HT
**Q**	BWT,WT,PNA,GA,PMA
**VP**	BWT,WT,PNA, GA,PMA

CL : Clearance; BWT : Birth Weight; PNA : Post Natal Age; GA : Gestational Age; PMA : Post Menstrual Age; WT : Weight; HT : Height; SCr : Serum Creatinine; CrCl : Creatinine Clearance; VC: Volume of Central Compartment; Q : Inter Compartmental Clearance; VP : Volume of Peripheral Compartment;

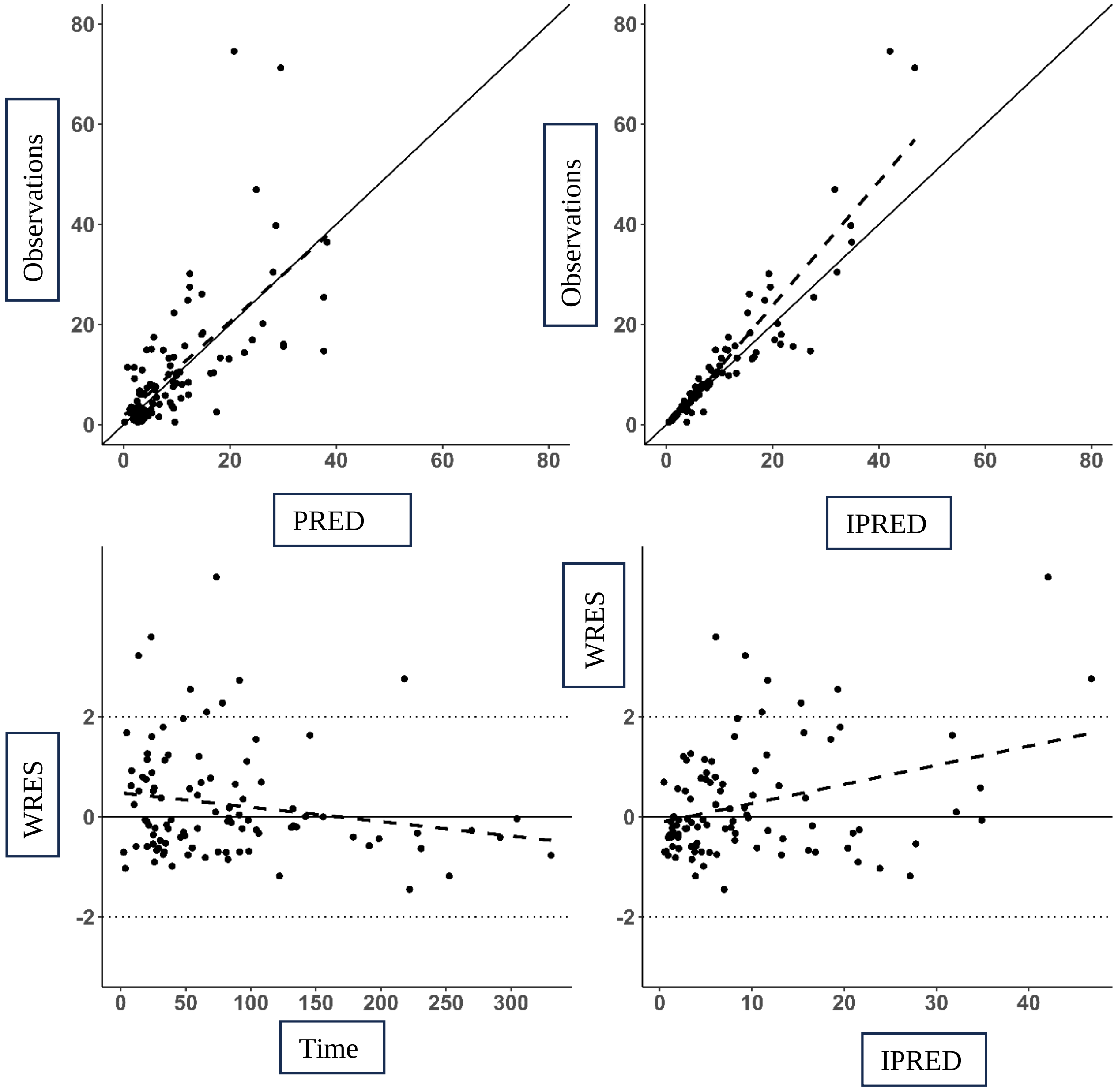

Supplementary Figure S1: Goodness of fit plots of base model. Solid line represents the identity line, while the dashed line illustrates the trend.
